# 2139. Immunogenicity, reactogenicity and safety of a Respiratory Syncytial Virus prefusion F (RSVPreF3) candidate vaccine co-administered with the seasonal quadrivalent influenza vaccine in older adults

**DOI:** 10.1093/ofid/ofac492.1759

**Published:** 2022-12-15

**Authors:** Reynaldo Chandler, Nathali Montenegro, Cecilia Llorach, Dean Quinn, Lorena Noriega-Aguirre, Mohammed Bensellam, Nathalie De Schrevel, Sherine Kuriyakose, Axel Lambert, Aurélie Olivier, Veronica Hulstrøm

**Affiliations:** CAENSA clinical trials, Panamá city, Panama, Panama; Centro de Vacunación e Investigación CEVAXIN S.A., Avenida México, Panamá city, Panama, Panama; Unidad Local de Atención Primaria de Salud de San Cristóbal, Caja de Seguro Social, Panamá; Instituto de Investigaciones Científicas y Servicios de Alta Tecnología AIP (INDICASAT AIP), Panamá, Panama City, Panama, Panama; P3 Research, Wellington, Wellington, New Zealand; Centro de diagnóstico y tratamiento de enfermedades respiratorias, CEDITER, Panamá city, Panama, Panama; GlaxoSmithKline Biologicals, Wavre, Brabant Wallon, Belgium; GSK, Rixensart, Belgium, Rixensart, Brabant Wallon, Belgium; GlaxoSmithKline, Bangalore, Karnataka, India; GlaxoSmithKline Biologicals, Wavre, Brabant Wallon, Belgium; GlaxoSmithKline Biologicals, Wavre, Brabant Wallon, Belgium; GSK, Wavre, Belgium, Wavre, Brabant Wallon, Belgium

## Abstract

**Background:**

Respiratory syncytial virus (RSV) can cause severe respiratory disease in older adults (OAs). However, there is no approved vaccine against RSV disease in OAs. Co-administration of vaccines against RSV and influenza could be considered given their overlapping seasonality. Here, we assessed the immunogenicity, reactogenicity and safety of an RSV prefusion F Older Adult (RSVPreF3 OA) investigational vaccine when co-administered with the seasonal quadrivalent influenza vaccine (FLU-QIV) in OAs.

**Methods:**

In this Phase 3, open-label, controlled, multi-country study (NCT04841577), OAs aged ≥ 60 years recruited in New Zealand, Panama and South Africa were randomized 1:1 to receive either RSVPreF3 OA and FLU-QIV simultaneously on day 1 (Co-Ad group), or FLU-QIV on day 1 and RSVPreF3 OA on day 31 (Control group). The co-primary objectives were to demonstrate the non-inferiority of i) RSVPreF3 OA in terms of RSV-A neutralizing antibody geometric mean titers (GMT) ratio and ii) FLU-QIV in terms of hemagglutinin inhibition antibody GMT ratio for each Flu strain when co-administered versus when administered alone. Blood samples were collected from all participants pre vaccination and 1 month post vaccination. Non-inferiority was demonstrated if the upper limit (UL) of the 95% confidence interval (CI) of the group GMT ratio (Control/Co-Ad) was ≤ 1.5. Secondary descriptive outcomes included reactogenicity and safety.

**Results:**

885 participants received at least 1 dose of the study interventions. Of these, 838 were included in the per protocol set at 1 month post vaccination. The demographic characteristics of the participants were similar across groups. The study co-primary objectives were met; for both vaccines, the UL of the 95% CI of the GMT ratio was ≤ 1.5 (Table 1). The observed safety events were balanced between Co-Ad and Control groups (Table 2). The reactogenicity profile of the Co-ad group compared with the Control group was driven by the RSVPreF3 OA investigational vaccine.

**Figure:**
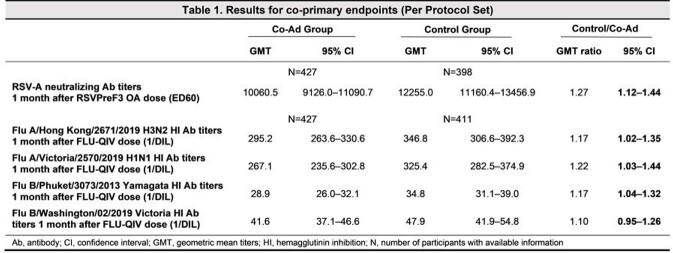

**Figure:**
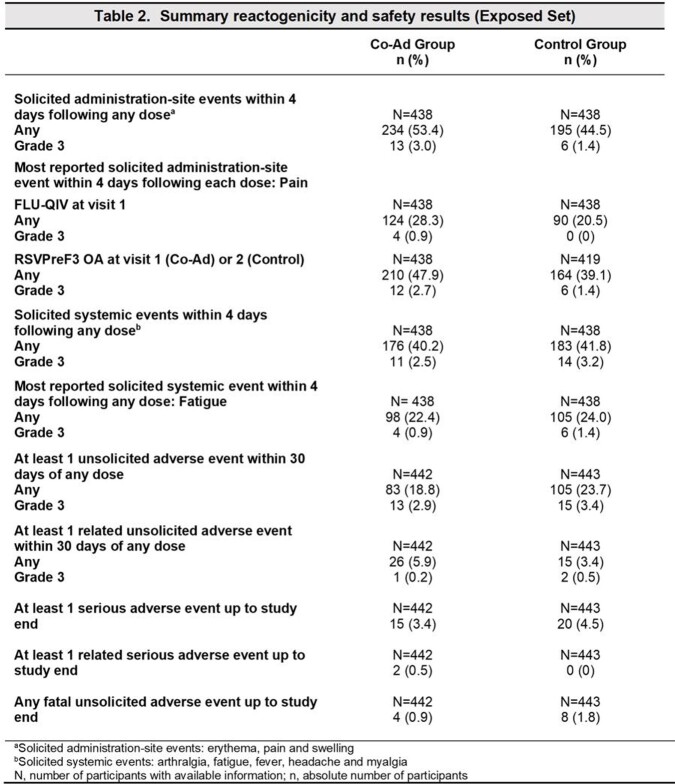

**Conclusion:**

Our results support simultaneous seasonal vaccination with RSVPreF3 OA and FLU-QIV in adults ≥ 60 years, as co-administration elicits a statistically non-inferior immune response to the administration of each vaccine alone, with no safety concerns identified.

**Disclosures:**

**Nathali Montenegro, MD**, GSK: Support for the present abstract/study **Lorena Noriega-Aguirre, MD, ScD**, Astra Zeneca: Advisor/Consultant|Astra Zeneca: Honoraria|Astra Zeneca: Support for attending meetings and/or travel|Boehringer Ingelheim: Honoraria|Boehringer Ingelheim: Support for attending meetings and/or travel|GSK: Advisor/Consultant|GSK: Support for the present abstract/study|Novartis: Honoraria **Mohammed Bensellam, PhD**, GlaxoSmithKline Biologicals SA: Agency worker on assignment at GSK **Nathalie De Schrevel, PhD**, GlaxoSmithKline Biologicals SA: Employee|GlaxoSmithKline Biologicals SA: Ownership Interest **Sherine Kuriyakose, MSc**, GSK: GSK Employee **Axel Lambert, MSc**, GSK: GSK Employee **Aurélie Olivier, PhD**, GlaxoSmithKline Biologicals SA: I’m an employee of GSK Biologicals|GlaxoSmithKline Biologicals SA: Ownership Interest **Veronica Hulstrøm, PhD MD**, GlaxoSmithKline Biologicals SA: Employee.

